# Neurodevelopmental outcomes following hematopoietic cell transplantation for patients with severe combined immunodeficiency (SCID): A PIDTC study

**DOI:** 10.70962/jhi.20250163

**Published:** 2025-12-17

**Authors:** Virdette L. Brumm, Sharon A. Kidd, Brent R. Logan, Farheen Chunara, Jennifer Heimall, Linda M. Griffith, Donald B. Kohn, Lauren Sanchez, Jeffrey J. Bednarski, Caridad Martinez, Mark Vander Lugt, Neena Kapoor, Nicola Wright, Barbara Spitzer, Joseph H. Oved, Sharat Chandra, Deepak Chellapandian, Christen L. Ebens, Aleks Petrovic, Ahmad Rayes, Hilary L. Haines, Hannah Lust, Hannah-Lise Tirado Schofield, Lauren Christopher, Lynnette L. Harris, Lisa Forbes Satter, Lauri Burroughs, Christopher C. Dvorak, Elie Haddad, Jennifer W. Leiding, Rebecca A. Marsh, Luigi D. Notarangelo, Sung Yun Pai, Michael A. Pulsipher, Jennifer M. Puck, Morton J. Cowan, Ami J. Shah

**Affiliations:** 1 Division of Pediatric Allergy, Immunology, and Blood and Marrow Transplantation, Department of Pediatrics, University of California San Francisco School of Medicine and UCSF Benioff Children’s Hospital, San Francisco, CA, USA; 2 Division of Biostatistics, Medical College of Wisconsin, Milwaukee, WI, USA; 3 Center for International Blood and Marrow Transplant Research, Milwaukee, WI, USA; 4 Division of Allergy and Immunology, Department of Pediatrics, Perelman School of Medicine at the University of Pennsylvania, Philadelphia, PA, USA; 5 Division of Allergy, Immunology and Transplantation, National Institute of Allergy and Infectious Diseases, National Institutes of Health, Bethesda, MD, USA; 6 Microbiology, Immunology & Molecular Genetics, University of California Los Angeles, Los Angeles, CA, USA; 7 Division of Hematology and Oncology, Department of Pediatrics, Washington University School of Medicine, St. Louis, MO, USA; 8 Center for Cell and Gene Therapy, Baylor College of Medicine, Texas Children’s Hospital, Houston, TX, USA; 9 Blood and Marrow Transplant Program, University of Michigan, Ann Arbor, Ann Arbor, MI, USA; 10 Transplantation and Cellular Therapy Section, Cancer and Blood Diseases Institute, Children’s Hospital Los Angeles, Keck School of Medicine, University of Southern California, Los Angeles, CA, USA; 11 Department of Pediatrics, University of Calgary, Calgary, Canada; 12 Children’s Cancer Institute, Hackensack University Medical Center, Hackensack, NJ, USA; 13 Stem Cell Transplantation and Cellular Therapy, MSK Kids, Memorial Sloan Kettering Cancer Center, New York, NY, USA; 14 Bone Marrow Transplantation and Immune Deficiency, Cincinnati Children’s Hospital Medical Center, Cincinnati, OH, USA; 15 Center for Cell and Gene Therapy for Non-Malignant Conditions, Johns Hopkins All Children’s Hospital, St Petersburg, FL, USA; 16 Division of Blood and Marrow Transplantation and Cellular Therapy, Department of Pediatrics, University of Minnesota, Minneapolis, MN, USA; 17 Fred Hutchinson Cancer Center, University of Washington School of Medicine and Seattle Children’s Hospital, Seattle, WA, USA; 18 Pediatric Immunology and Hematopoietic Cell Transplantation/Cellular Therapy Program, Primary Children’s Hospital, University of Utah, Salt Lake City, UT, USA; 19 Division of Pediatric Hematology and Oncology and Bone Marrow Transplant, University of Alabama at Birmingham, Birmingham AL, USA; 20 Division of Pediatric Hematology, Oncology, Stem Cell Transplantation, Ann & Robert H. Lurie Children’s Hospital of Chicago, Northwestern University Feinberg School of Medicine, Chicago, IL, USA; 21 Division of Oncology, Cellular Therapy and Transplant Section, Children’s Hospital of Philadelphia, Philadelphia, PA, USA; 22 Division of Developmental Medicine, University of California San Francisco, San Francisco, CA, USA; 23 Department of Pediatrics, Psychology Service Baylor College of Medicine, Houston, TX, USA; 24 Division of Immunology, Allergy and Retrovirology, Department of Pediatrics, Center for Human Immunobiology, Texas Childrens Hospital, Houston, TX, USA; 25 Department of Pediatrics, Department of Microbiology, Immunology and Infectious Diseases, Center de Recherche Azrieli du Sainte-Justine University of Montreal, Montreal, Canada; 26 Laboratory of Clinical Immunology and Microbiology, National Institute of Allergy and Infectious Diseases/National Institute of Health, Bethesda, MD, USA; 27 Immune Deficiency - Cellular Therapy Program, Center for Cancer Research, National Cancer Institute/ National Institute of Health, Bethesda, MD, USA; 28 Division on Hematology Oncology, Intermountain Primary Children’s, Huntsman Cancer Institute, Spencer Fox Eccles School of Medicine at the University of Utah, Salt Lake City, UT, USA; 29 Department of Pediatrics, Pediatric Hematology Oncology, Stem Cell Transplantation and Regenerative Medicine, Stanford University, Palo Alto, CA, USA

## Abstract

Hematopoietic cell transplantation (HCT) is a potentially curative treatment for severe combined immunodeficiency (SCID). Since the initiation of newborn screening (NBS), survival rates have improved significantly, but the impact of HCT upon neurodevelopment for patients with SCID requires more investigation. We performed a cross-sectional study of subjects with SCID in North America to assess the impact of NBS, transplant conditioning regimen, and genotype on neurodevelopmental outcomes after HCT. 69 subjects with SCID from 17 PIDTC centers (excluding those with ADA deficiency), ages 6–16 years, received comprehensive standardized neurodevelopmental testing of cognitive, behavioral, and emotional function. Compared with the normative population, our subjects performed in the average range. We found no impact of NBS, chemotherapy conditioning, or genotype. Multivariate analysis revealed a significant decrease in IQ in subjects whose families earned <$50,000 per year. We recommend that children treated by HCT for SCID be monitored with periodic cognitive and behavioral assessments for deficits that could potentially impact long-term ND outcomes.

## Teaser

We evaluated 69 subjects with SCID to assess the neurodevelopmental outcomes following HCT. Compared with the normative population, our subjects performed in the average range. There was no impact of newborn screening, conditioning, or genotype. Families earning <$50,000 had poorer outcomes. Our study provides the most substantial, comprehensive analysis of ND outcomes for SCID.

## Introduction

Severe combined immunodeficiency (SCID) is an inborn error of immunity due to mutations in a variety of genes causing the absence of T cell immunity and nonfunctioning or absent B cells, resulting in life-threatening infections (1). With newborn screening (NBS) for SCID, patients can now be diagnosed early in infancy and receive definitive therapy prior to developing severe infections (2). Early diagnosis can also lead to early definitive therapy to establish a hematopoietic stem cell population capable of generating functional T cells and, ideally, B cells, by allogeneic hematopoietic cell transplantation (HCT) or gene therapy (GT), although patients with adenosine deaminase (ADA)–deficient SCID have also been managed with chronic enzyme replacement therapy (1).

The Primary Immune Deficiency Treatment Consortium (PIDTC), a member of the Rare Diseases Clinical Research Network sponsored by National Institutes of Health (NIH), previously reported that patients with SCID who had infections at the time of HCT had decreased overall survival compared with those who did not (3, 4). Since the adoption of NBS for SCID in the USA and Canada beginning in 2008 (2, 5), the 5-year overall survival for individuals with SCID has improved from 72% (1982–2009) to 87% (2010–2018). This increase in survival was achieved due to younger age at which patients received definitive treatment and freedom from infections at the time of transplant (6). Despite recent improvement in overall survival after HCT, patients with SCID may continue to experience chronic and late effects that impact their health (7).

Neurodevelopmental (ND) studies for patients with serious, nonmalignant diseases, including SCID, have been limited due to the rarity of these diseases, the wide range of ages of survivors (including adults), and the variability of patient presentations and treatments. For SCID, published studies performed before NBS was introduced were often limited to single institutions, a variety of different age ranges, and limited test measures (8, 9, 10), or included other primary immune disorders in addition to SCID (10). Lower intelligence quotient (IQ) was reported to be associated with consanguinity, diagnosis of ADA deficiency, and severe clinical infections prior to transplantation (10, 11).

We performed a cross-sectional study to assess the impact of NBS, genotype, and conditioning for HCT on ND outcomes for children with SCID. Our study is unique in that we were able to perform the same comprehensive test battery on all subjects within the age range specified for investigation.

## Results

**Figure 1.**
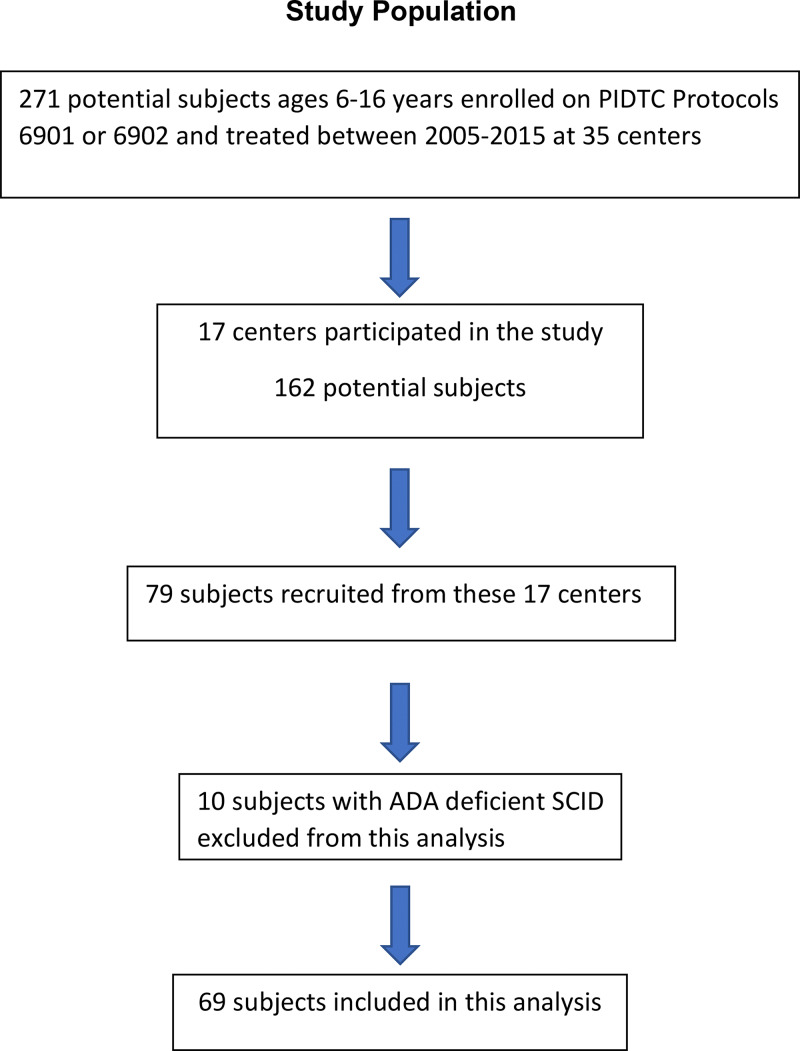
Experimental schema.

There were 271 subjects who were eligible for this study, but 79 subjects at 17 sites were enrolled and tested. Of these, 10 subjects had ADA-deficient SCID and were excluded (Fig. 1). The demographics of the 69 subjects analyzed in this ND study are shown in Table 1. The excess of males in the study reflects the known substantial proportion of X-linked SCID cases, which was not statistically different between the study population and the potentially eligible population.

**Table 1.** Subject characteristics

​	​	*n* (%)
Sex		
​	Male	49 (71.0%)
​	Female	20 (29.0%)
Age		
​	Median	9 years
​	Range	6–16 years
Race/ethnicity		
​	American Indian/Alaska native (non-Hispanic)	4 (6.45%)
​	Asian/Pacific Islander	9 (14.52%)
​	Black	3 (4.84%)
​	Hispanic (any race)	19 (30.65%)
​	White/non-Hispanic	27 (43.55%)
​	Unknown	7 (6.4%)
Mutation		
​	DCLRE1C	4 (5.8%)
​	IL2RG/or JAK3	34 (49.3%)
​	IL7R/or CD3D	10 (14.5%)
​	RAG1/2	11 (15.9%)
​	Unknown	10 (14.5%)
Trigger for diagnosis		
​	FH	12 (17.4%)
​	Infection/or clinical symptoms	24 (34.8%)
​	NBS	33 (47.8%)
Donor type		
​	Autologous (GT)	3 (4.4%)
​	HLA matched sibling	4 (5.8%)
​	HLA matched unrelated/other non-sibling relative	25 (36.2%)
​	HLA mismatched relative	23 (33.3%)
​	HLA mismatched unrelated	14 (20.3%)
Type of conditioning		
​	None/immunosuppressive therapy alone	38 (55.1%)
​	Reduced intensity/myeloablative	31 (44.9%)

There was a statistical difference in the conditioning regimen between our study population and the potentially eligible population (Table S1). The percentage of subjects in our study who did not receive conditioning or received immunosuppression (IS) only (none/IS) was more than in the eligible population (50.6% *n* = 40 vs 34.6%, *n* = 56; P < 0.001). The percentage of subjects in our study who received reduced intensity or myeloablative conditioning (RIC/MAC) was less than in the eligible population (49.4%, *n* = 39 vs. 65.4%, *n* = 106, P < 0.001).

### Cognitive outcomes

**Figure 2.**
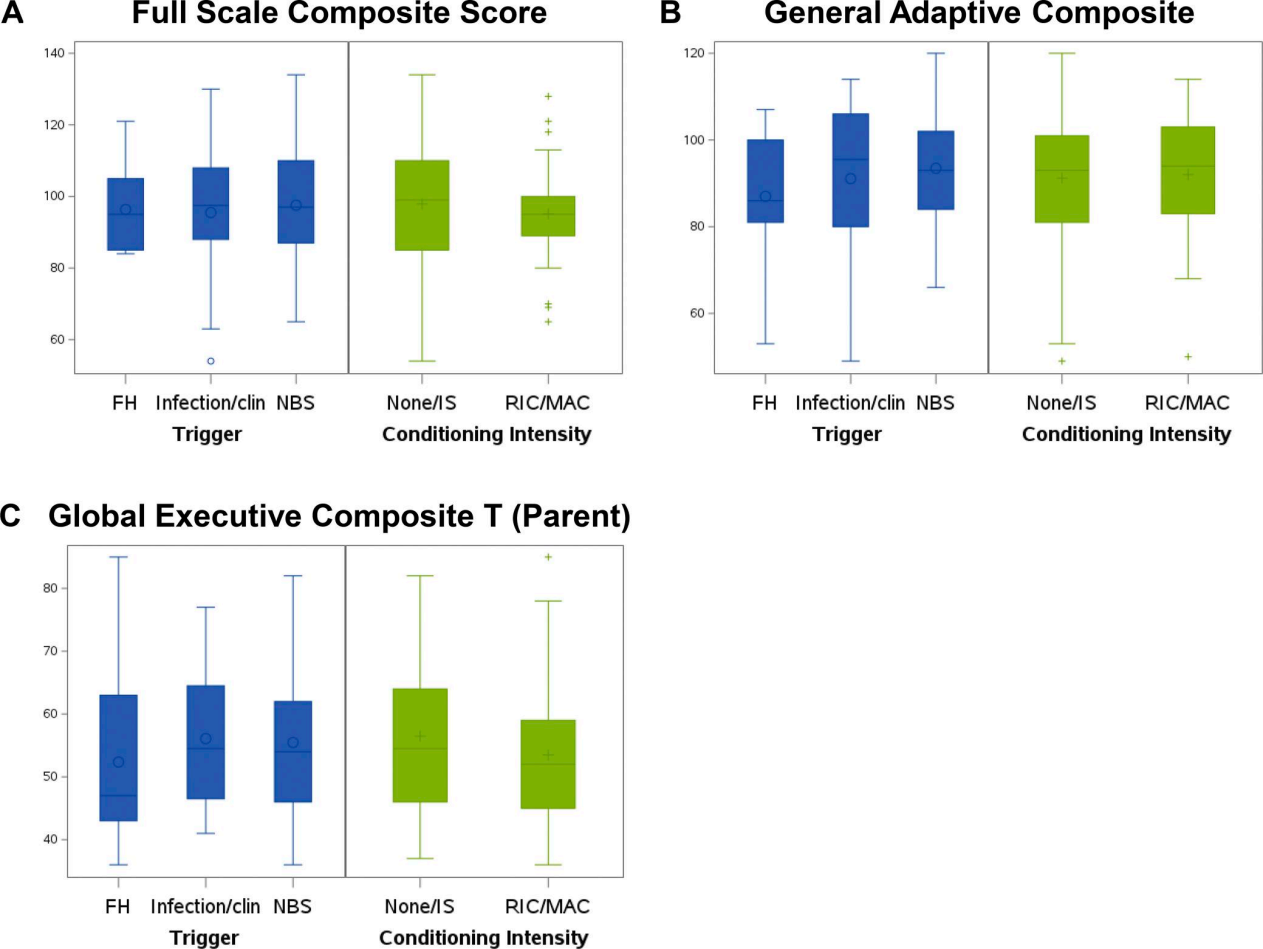
**Overall cognitive scores. (A)** The differences of the full composite score, global executive score, and general adaptive score in two comparison groups. **(B)** Blue bars show the difference between subjects and how the trigger for their diagnosis of SCID impacted outcomes. Infection/clin, infection or clinical symptoms. **(C)** Green bars show the difference between those who received no chemotherapy or IS alone (none/IS) vs. those receiving either reduced intensity chemotherapy vs. myeloablative condioning (RIC/MAC).

Table 2 contains the mean scores for the ND tests that were performed on the study cohort in comparison to the normative population. For cognitive testing, based on the Wechsler Intelligence Scale for Children (WISC-V) Full-Scale Intelligence Quotient (FSIQ), the mean IQ (96.94) for the study cohort was in the average range (M = 100, SD 15) (Table 2). We assessed whether specific clinical factors, including conditioning, and trigger for diagnosis impacted FSIQ (Fig. 2 A), adaptive function (Fig. 2 B), or behavior outcomes (General Executive Composite Score) (Fig. 2 C). There was no difference (P = 0.53) in the mean IQ between patients who received none/IS (normative mean [M] = 97.89, standard deviation [SD] = 18.91, *n* = 38) vs. MAC/RIC (M = 95.14, SD = 14.79, *n* = 31) (Table 3). There was also no difference (P = 0.95) in the mean IQ between subjects identified by NBS (M = 98.88, SD 19.55, *n* = 33), family history (FH) (M = 97.09, SD 13.35, *n* = 12), or clinical illness (M = 98.88, SD 19.55, *n* = 33) (Table 4). Genotype did not have an impact on mean IQ. The IQs for those with genotypes *IL2RG*, *JAK3*, *IL7R*, *RAG1/2*, and unknown were within the average range, while IQs of those with *DCLRE1C* defects (*n* = 4) had a low average IQ (M = 88.85, SD = 7.54), although not statistically different from the normative population. Notably, 7.81% (95% confidence interval [CI] 2.59%), of the study cohort (*n* = 5) (Table 5) fell in the extremely low range (IQ < 69) compared with the normative population in which only 2% have scores in the extremely low range (P < 0.0004).

**Table 2.** Overall scores for individual ND tests

Test, M and SD (*n* for study population tested)		Study population mean test score (SD)
**Cognition outcome testing**		
WISC-V (M = 100, SD 15) (*n* = 69)		
​	FSIQ	96.64 (17.09)
​	Verbal comprehension index	98.34 (19.34)
​	Visual spatial index	99.05 (15.49)
​	Fluid reasoning	98.69 (16.80)
​	Working memory	94.28 (16.08)
​	Processing speed index	94.88 (16.81)
Delis–Kaplan Executive Function System (D-KEFS) (M = 10, SD 3) (*n* = 66)		
​	Number letter switching	8.52 (3.47)
​	Tower total achievement	9.79 (2.20)
California Verbal Learning Test (Children’s version [CVLT-C]) delayed recall (M = 0, SD 1) (*n* = 63)		−0.10 (1.16)
Beery Visual-Motor Integration (M = 100, SD 15) (*n* = 67)		84.27 (15.46)
Adaptive Behavior Assessment System (ABAS-3) (M = 100, SD 15) (*n* = 64)		
​	General adaptive composite	91.55 (15.08)
​	Conceptual domain	90.32 (14.79)
​	Social domain	94.18 (15.28)
​	Practical domain	94.12 (16.00)
**Emotional and behavioral outcome testing**		
BRIEF-2 (M = 50, SD 10) (*n* = 47)		
​	Global executive composite	55.18 (12.14)
​	Behavior regulation index	53.10 (11.57)
​	Emotional regulation index	55.33 (12.86)
​	Cognitive regulation index	53.96 (11.27)
BASC-3 (M = 50, SD 10) (*n* = 66)		
​	Internalizing	51.65 (10.90)
​	Externalizing	50.50 (10.30)
​	Behavior symptoms index	52.24 (10.00)
​	Attention problems	53.82 (11.18)
​	Hyperactivity	52.55 (10.92)
Beck Youth Inventories (M = 50, SD 10) (*n* = 53)		
Depression inventory (*n* = number of patients who tested in that category)		46.04 (8.68)
​	Average (*n* = 44)	​
​	Mildly elevated (*n* = 5)	​
​	Moderately elevated (*n* = 3)	​
​	Extremely elevated (*n* = 1)	​
Anxiety inventory (*n* = number of patients who tested in that category)		47.94 (10.01)
​	Average (*n* = 41)	​
​	Mildly elevated (*n* = 7)	​
​	Moderately elevated (*n* = 3)	​
​	Extremely elevated (*n* = 2)	​

**Table 3.** Univariate comparison of primary neurocognitive measures based on conditioning regimens

Test	None/IS *n* = 38 mean (SD)	RIC/MAC *n* = 31 mean (SD)	Total mean (SD)	P value
FSIQ	97.89 (18.91)	95.14 (14.79)	96.64 (17.09)	0.53
General adaptive composite score	91.22 (15.74)	92.00 (14.40)	91.55 (15.08)	0.84
Global executive composite T score (parent)	56.47 (12.04)	53.48 (12.27)	55.18 (12.14)	0.32
Internalizing problems T score	52.57 (12.24)	50.48 (8.98)	51.65 (10.90)	0.44
Externalizing problems T score	51.46 (11.84)	49.28 (7.96)	50.50 (10.30)	0.38
Behavioral symptoms T score	52.82 (9.71)	51.52 (10.49)	52.24 (10.00)	0.61

**Table 4.** Univariate comparison of primary neurocognitive measures based on trigger for diagnosis

​	FH (*n* = 12) mean (SD)	Infection/clinical symptoms (*n* = 24) mean (SD)	NBS (*n* = 33) mean (SD)	Total (*n* = 69) mean (SD)	P value
FSIQ	96.36 (11.72)	95.50 (19.84)	97.55 (17.03)	96.64 (17.09)	0.91
General adaptive composite score	87.00 (15.78)	91.09 (18.04)	93.48 (12.45)	91.55 (15.08)	0.47
Global executive T score (parent)	52.36 (14.79)	56.08 (10.71)	55.47 (12.44)	55.18 (12.14)	0.70
Internalizing problems T score	52.09 (11.05)	54.79 (11.35)	49.06 (10.16)	51.65 (10.90)	0.15
Externalizing problems T score	51.18 (9.45)	50.42 (7.21)	50.32 (12.66)	50.50 (10.30)	0.97
Behavioral symptoms T score	53.27 (11.70)	53.92 (8.81)	50.58 (10.30)	52.24 (10.00)	0.45

**Table 5.** Frequency of clinically significant test scores^a^

Test	Frequency in clinically significant range (*n*)	Percent of subjects tested (*n* = patients tested)
FSIQ	5	7.81% (*n* = 64)
Global executive composite (patient)	3	13.6% (*n* = 22)
Global executive composite (parent)	10	14.93% (*n* = 67)
Visual motor integration	8	11.94% (*n* = 67)
Verbal comprehension	8	12.31% (*n* = 65)
Visual spatial	1	1.56% (*n* = 64)
Fluid reasoning	2	3.08% (*n* = 65)
General ability index	4	6.15% (*n* = 65)
Working memory	6	9.38% (*n* = 64)
Processing speed	5	7.81% (*n* = 64)
Internalizing problems	4	6.06% (*n* = 66)
Externalizing problems	2	3.0% (*n* = 66)
Behavioral symptoms	4	6.06% (*n* = 66)
Behavioral regulation (patient)	4	18.18% (*n* = 22)
Behavioral regulation (parent)	8	11.94% (*n* = 67)
Emotion regulation (patient)	4	18.18% (*n* = 22)
Emotion regulation (parent)	10	14.93% (*n* = 67)
Cognitive regulation Index (patient)	2	9.09% (*n* = 22)
Cognition regulation Index (parent)	8	11.94% (*n* = 67)
Attention problems	5	7.58% (*n* = 66)
Hyperactivity	9	13.64% (*n* = 66)

a Clinically significant low test scores are defined as <2 SD below the mean.

Specific cognitive domains of ND functioning had not been previously evaluated in subjects with SCID (8, 9, 10); therefore, tests of executive function, verbal memory, and visual motor integration were assessed (Table 2). Although the mean scores of these specific cognitive domains were in the average range for most subjects, a minority of subjects had very low test scores (Table 5). In the domain of executive function, which includes higher level cognitive abilities, such as working memory, planning, organization, cognitive flexibility, impulse control, and emotional control (D-KEFS Trail Making Switching; D-KEFS Tower), the mean scores were in the average range (D-KEFS Switching 8.52 and Tower 9.79) (M = 10, SD = 3) (Table 2). In the working-memory domain, although the mean score of the cohort was in the average range, 9.52% (*n* = 6) (95% CI 3.58%, 19.59%) had low scores compared with 2% of the normative population (P < 0.0001). In the visual-motor domain, 11.94% (*n* = 8) (Table 5) subjects tested in the extremely low range (CI = 5.30%, 22.18%, P < 0.0001).

### Adaptive behavior

Adaptive abilities consist of skills used to function in daily life and school. Compared with the normative population, parents reported scores in the average range (91.55) (Table 2); however, 7.81% (95% CI 2.59%, 17.30%) of the study cohort (*n* = 5) fell in the low range compared with 2% of the normative population (P = 0.0004). Analysis of specific SCID risk factors (trigger for diagnosis, conditioning regimen, and genotype) showed no difference in the adaptive skills between patients who had received RIC/MAC (M = 92.00, SD = 14.40) compared with none/IS (M = 91.22, SD = 15.74) (Fig. 2 B). Adaptive skills in patients with certain genotypes (*IL7R/CD3D* M = 88.20; unknown mutations M = 85.78) were in the low average range but were not statistically different from normative values. The trigger for diagnosis had no impact on adaptive skills assessed in this study. In-depth analysis of specific domains of adaptive function, including conceptual, social, and pragmatic skills, indicated that the mean scores in all three domains were in the average range compared with the normative population (Table 4).

### Behavioral outcomes

The behavioral outcomes of the study cohort consisted of both parent- and patient-report questionnaires. Parents completed two questionnaires: (1) a composite measure of the behavioral aspects of their child’s executive function (i.e., emotional regulation, attention problems, and hyperactivity [Behavior Rating of Executive Function-2, BRIEF-2]) and (2) an inventory of social-emotional functioning (Behavior Assessment System for Children, BASC-3). Subjects completed inventories assessing emotional adjustment based on the presence of depression and anxiety symptoms. On the parent-report inventory (BRIEF-2) assessing behavioral aspects of their child’s executive function (e.g., impulse control and emotional regulation), the mean score was in the average range (Table 2). These behaviors were not associated with conditioning regimen, trigger for diagnosis, or genotype (Fig. 2 C).

**Figure 3.**
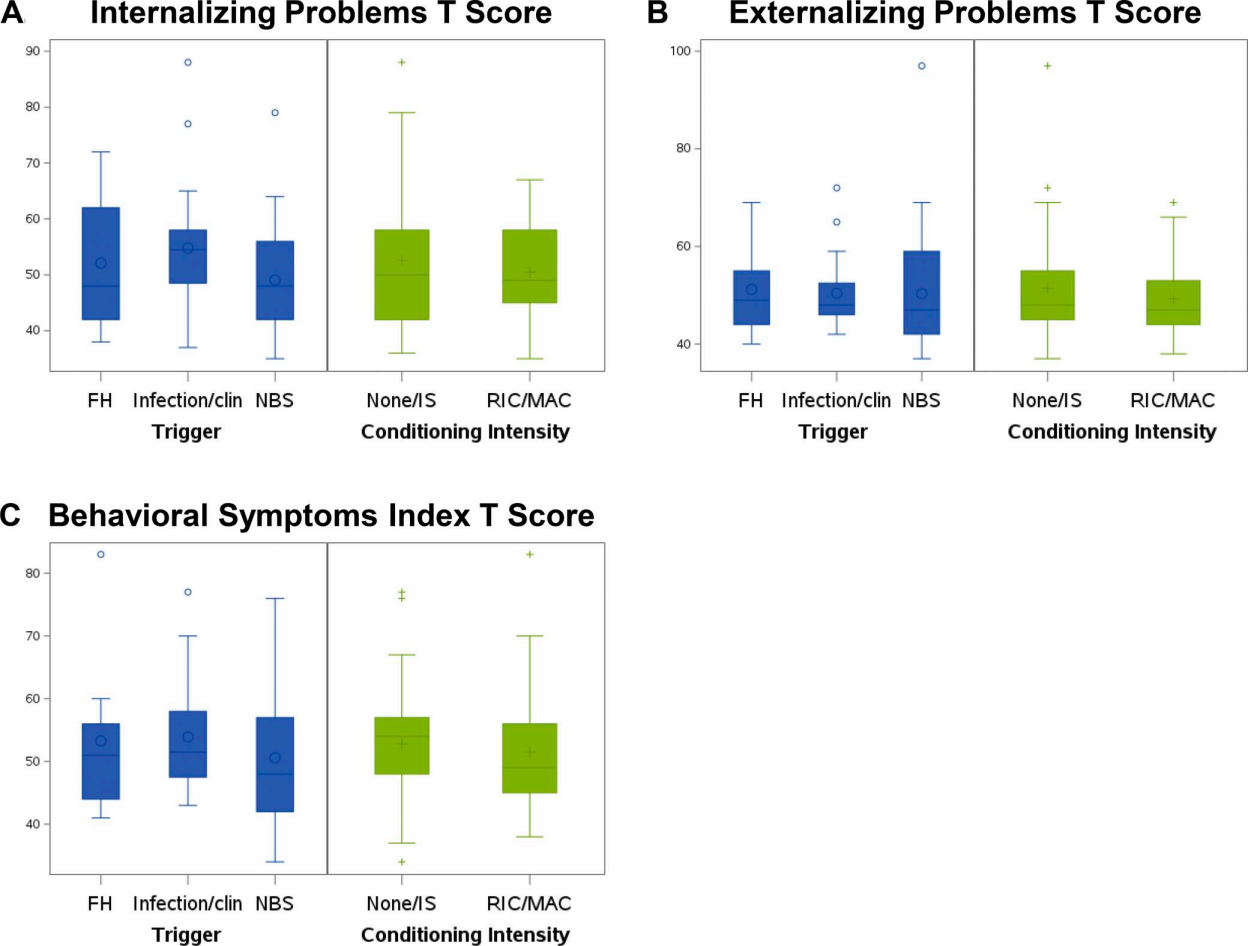
**Behavior symptoms. (A)** The differences of the internalizing and externalizing problems and behavior symptoms in two comparison groups. **(B)** Blue bars show the difference between subjects and how the trigger for their diagnosis of SCID impacted outcomes. Infection/clin, infection or clinical symptoms. **(C)** Green bars show the difference between those who received no chemotherapy or IS alone (none/IS) vs. those who received either reduced intensity chemotherapy vs. myeloablative condioning (RIC/MAC).

The results of the internalizing, externalizing, and overall behavioral symptoms are shown (Fig. 3, A and B). Compared with the normative population, 14.93% (*n* = 10) subjects had significant problems with executive function (standard score [SS] = >70) compared with 2% of the normative population. More specifically, 8 subjects (11.94%) had more behavioral regulation problems compared with the normative population, and 10 subjects (14.93%) had emotional regulation problems (Table 5).

A second parent questionnaire (BASC-3) provided data on the cohort’s emotional-behavioral adjustment as reflected by internalizing behavior (e.g., depression and anxiety), externalizing behavior (hyperactivity and conduct problems), and behavior symptoms (attention problems and atypicality). Four subjects (6.06%) had internalizing behavioral symptoms in the clinically significant range, and two subjects (3.03%) had externalizing behavioral symptoms (Table 5). Scales measuring attention and hyperactivity were in the average range.

Subjects completed inventories measuring the presence of depression and anxiety symptoms. 53 subjects ages 7 and older completed a self-report measure of depression. Of these, 44 subjects (83%) reported none to minimal depressive symptoms, 5 (9.43%) reported a mild level of symptoms, 3 (5.66%) reported a moderate level of symptoms, and 1 subject reported an extremely increased level of symptoms (Table 2). We also compared the incidence of depression (BDI-Y score) among those who had a history of chronic graft versus host disease (GVHD) (46.33, *n* = 14) versus those who did not have chronic GVHD (46.18, *n* = 54), P = 0.96. These 53 patients also completed a self-reported measure of anxiety symptoms (Beck Youth Inventories). Most subjects had minimal to no symptoms of anxiety, *n* = 41 (77.36%). However, seven subjects (13.21%) had mild symptoms, four (5.66%) moderate symptoms, and two subjects (3.77%) had extremely elevated symptoms (Table 2).

### Co-occurring deficits

22 of the 69 subjects in the study had a low score on one or more of the neurodevelopment measures: 13 subjects had one low score, 6 subjects had two low scores, and 3 subjects had three low scores (Table 5). It is well established that individuals with low intellectual ability are expected to have more low scores (12). Of the 22 subjects who had at least one low score, 5 subjects had a low IQ score based on the WISC-IV. Of those, 3 had one additional low test score, and 2 subjects had two additional low test scores. We examined the variables for these five subjects, as compared with the entire cohort, to determine if there were any factors that could have contributed to their low scores. Four of these five subjects had an infection prior to receiving their definitive therapy. Of note, one of the subjects received a matched unrelated donor (MUD) hematopoietic cell transplant following two unsuccessful autologous unconditioned GT procedures. Prior to the MUD transplant, this patient had *Escherichia coli* meningitis and a seizure disorder (Table S2).

### Role of external factors on neurocognitive function

To investigate the potential impact of external factors upon ND, we performed multivariate regression models for socioeconomic factors. We found that self-reported household income had a dose response relationship with the WISC composite score. (Table 6). Subjects of the 15 families whose annual income was <$50,000 had significantly lower overall cognitive function (P = 0.001) compared with those whose family incomes were higher. We assessed whether language spoken at home may have contributed to the global executive composite T score (BRIEF-2). There were 27 patients who reported speaking a language other than English (or French, for Canadian families). These individuals demonstrated a significant decrease in the executive composite score (P = 0.008) (Table 6).

**Table 6.** Impact of external factors upon ND tests

​			
**Impact of total annual household income upon overall FSIQ (WISC-V)**			
Income level	*n*	Estimate (95% CI)	P value
>$150,000	13	Reference value	​
$50,000–$150,000	28	−14.73 (−25.73, −3.73)	0.010
<$50,000	15	−21.29 (−33.68, −8.89)	0.001
Missing	6	na	​
Prefer not to answer	4	na	​
**Impact of language other than English spoken at home upon global executive composite T score (BRIEF-2, parent)**			
Response	*n*	Estimate (95% CI)	P value
No	40	Reference value	​
Yes	27	−8.11 (−14.04, −2.18)	0.008

na, not applicable.

## Discussion

There have been extensive studies performed on ND outcomes following HCT for malignant diseases (13, 14, 15). However, patients with malignant disorders have typically been exposed to high doses of chemotherapy and may have received intrathecal chemotherapy or total body or cranial irradiation. Therefore, transplant outcomes for patients with malignant diseases cannot be compared with transplant outcomes for those with SCID. The impact of HCT on ND outcomes in patients with SCID has not been fully investigated. Most previous ND studies were from single institutions with limited numbers of subjects and a limited test battery due to the different ages tested (8, 9, 10). Previous research showed an increased risk of long-term cognitive difficulties and associated emotional and behavioral difficulties in patients with SCID treated with HCT (10).

Our study improves on previous investigations because of the comprehensive standardized test battery administered to subjects to evaluate domains of brain function not previously evaluated, including executive function, memory, visual-motor integration, and depression. In addition, we were able to include several test sites to allow for a variety of different conditioning regimens and types of transplants. Finally, we were able to assess the impact of diagnosing patients by NBS or by infections or other clinical symptoms.

In our cohort, most children who had undergone HCT or GT for SCID performed in the average range on cognitive measures, including IQ, memory, executive function, and visual-motor integration, as compared with the normative population. We did not find a significant impact of clinical variables, including trigger for diagnosis, genotype, or conditioning regimen, on ND outcomes. At least one low test score was observed in 22 of 69 subjects, and 5 subjects had more than one low test score. The frequency of single low scores in the study sample is similar to the normative population and not considered clinically significant. However, the prevalence of multiple low scores may be related to low IQ and an indicator of possible neurocognitive dysfunction (16). Although we could not detect factors that were associated with the low scores in the five subjects who had multiple low scores (Table S2), a larger study might reveal additional factors contributing to low scores.

It has been well established that the family’s socioeconomic status (SES) ranks among the strongest correlates of health and well-being in the general population (17). Previous research has shown that socioeconomically disadvantaged children were more likely to have ND problems (18). Our study also showed an association between lower family income and lower overall IQ. Although there are many factors that encompass SES that this study was unable to investigate, a larger study might identify contributors to lower ND function, such as parental education level or food insecurities.

In assessment of emotional and behavioral outcomes in this study, 9 subjects of the 53 tested reported mild to elevated depressive symptoms and anxiety. For most mental health disorders, including depression and anxiety, environmental stressors play a major role. This study was performed between 2020 and 2023, which was the height of the COVID-19 pandemic. Although the COVID-19 pandemic was a negative stressor to much of the population, there were other individuals who reported a positive impact with improved changes in their work life balance, family dynamics, and enhanced feelings of closeness (19). Loneliness is a strong correlate of depression and anxiety. It has been reported that adolescents, compared with younger children and older adults had disproportionate mental health effects due to COVID-19 secondary to unfavorable social changes such as school closures (20). A meta-analysis of studies assessing the impact of the COVID-19 pandemic upon the mental health of children showed an increase in depressive symptoms (21). Thus, it is not possible to determine the degree to which COVID-19 contributed to the observed results of symptoms of depression and anxiety on this population. Future studies should continue to monitor the mental health of children with SCID following HCT, including measures of depression and anxiety.

Our study has several limitations. First, the COVID-19 pandemic had a major impact on center participation. Due to the pandemic, many institutions were unable to open and/or perform nontherapeutic studies. As a result, only 17 of the 35 PIDTC sites that had enrolled participants aged 6–16 years who had been treated during 2005–2015 could participate (Fig. 1). Staffing and scheduling were limited, and many neuropsychologists were unavailable to perform testing in person. Second, from the 17 PIDTC centers that participated in this ND study, less than half of the potential subjects (48%) enrolled. Families with children with SCID were keenly aware of risks of infections and may not have wanted to travel to the hospital to participate in a nontherapeutic study. Also, of the potentially available subjects, there was a bias toward subjects who did not receive chemotherapy conditioning. We do not know the reasons for this difference. We thus remain cautious about these results and recognize that a larger study may detect differences not observed here.

In summary, the present study provides the most substantial and comprehensive analysis of ND outcomes for patients with SCID following HCT to date. This is particularly relevant for clinicians and families facing HCT for SCID due to concerns regarding chemotherapy. The in-depth assessment of cognition and behavior in the present study did not show an impact of pre-HCT conditioning on ND outcomes. Most children tested had normal ND outcomes, although low scores were identified in a few patients. In the context of ongoing brain development and potential deficits that may emerge, or resolve with maturation, we recommend that all patients with SCID have long-term monitoring of ND status to identify individuals who would benefit from additional academic and mental health support. Continued evaluation of treated patients is needed to advance our knowledge of the long-term sequelae of HCT on children with SCID.

The present study confirms and expands the conclusions of previous studies and demonstrates that the majority of SCID patients who receive HCT or GT have normal neurocognitive development. Moreover, our approach and test battery serve as a guide for comprehensive ND assessment in patient care and future studies.

## Materials and methods

### Subjects

Potential subjects were identified by their enrollment in PIDTC natural history protocols 6901 (prospective) or 6902 (retrospective) (https://ClinicalTrials.gov identifiers NCT01346150 and NCT01186913, respectively). De-identified, coded disease and transplantation-related data, including the trigger for diagnosis, genotype, and type of chemotherapy conditioning, were obtained for each participant from an electronic data base (3, 4, 5). This cross-sectional study, consisting of an ND assessment protocol combined with use of the natural history data, was approved by the PIDTC central institutional review board (IRB) at University of California, San Francisco and individual IRBs at participating PIDTC sites. Subjects with SCID who were 6–16 years of age and >5 years after HCT were invited to participate in this ND study. Informed consent was obtained from parents or guardians of subjects, and assent was obtained from subjects 7–16 years of age. The age range of 6–16 was chosen so that a consistent standard battery of tests could be used across centers. Fig. 3 shows the potential participants from PIDTC Protocols 6901 and 6902; 17 centers participated and 69 subjects received ND testing and are included in this analysis. PIDTC Protocols 6901 and 6902 enrolled and collected data for all sequential eligible children at each PIDTC center. Of the participating subjects, most received only one transplantation (*n* = 68); however, one subject received three different transplants (two GT products and one MUD transplant with MAC). This subject was classified within the MAC cohort. This ND study was limited by selection biases, including a center’s ability to participate in the ND study (due to, for example, availability of a neuropsychologist to perform testing) and the ability of a potential subject's family to travel to a participating center for completion of ND assessments in person. The characteristics of the resulting ND study population are shown in Table 1. Table S1 compares the participants included in this ND study to the population potentially eligible at centers performing ND testing. The study was conducted between January 2020 and July 2023. All SCID subject testing was conducted in English. Bilingual (Spanish/English) parents of subjects were included and completed inventories of behavioral and adaptive function in Spanish. Children who did not speak English, were deaf, or had trisomy 21, Duchenne muscular dystrophy, or nonverbal autism spectrum disorder were excluded. 10 children with ADA-deficient SCID were excluded from this analysis because ADA deficiency per se is known to cause neurocognitive impairments in some patients (11, 13).

The trigger for diagnosis was defined as the initial event leading to the diagnosis of SCID. Trigger categories were: NBS, FH, or clinical illness, including infection (6). Conditioning regimens were separated into two categories: None/IS only, or MAC/RIC, as previously described (3).

### Neurodevelopment testing

Consenting subjects completed a ND assessment at one of 17 participating PIDTC centers. ND evaluation was conducted or supervised by a qualified pediatric neuropsychologist. Testing procedures followed standard clinical guidelines with fixed test order and schedules to reduce effects of interference and fatigue. The test battery chosen for this study included standard tests that measure all areas of neurodevelopment. The test battery was administered using a variety of methods, including manual administration (i.e., pencil and paper), web-based on an iPad, and/or via internet-based test administration programs (Q Interactive). Test data were scored using standardized test manuals and computer-based, and/or internet-based scoring systems (Q-Global; PARiConnect).

Test scores were defined by the test instruments scoring instructions based on the normative population. Extremely low scores were defined as <2 SD below the standardized mean defined by each individual test.

### Measures

The test battery included age-appropriate measures of cognition in the domains of overall intellectual function, executive function, verbal memory, visual-perceptual skills, and information processing speed (Table 7). Behavioral and adaptive measures were completed by the parents/caregivers at the time of the patient’s study visit, or rarely by mail.

**Table 7.** Standardized ND test battery

Test (reference)	Domain/test description	Age of participants tested (6–16 potentially)/who completed testing
ABAS-3 (12)	Adaptive function includes the skills needed to effectively and independently care for oneself, respond to others, and meet the demands of home, school, and the community *The ABAS-3 measures the general adaptive composite (all skill areas), which includes:* *Conceptual domain: (communication, academic skill, and task completion)* *Social domain: (personal interaction and social responsibility)* *Practical domain: (personal and health needs at school and community)*	All ages, completed by parents only
Beck Youth Inventory (depression and anxiety) (22)	Emotional adjustment assess depression and anxiety *Clinical ranges are based on T scores from the standardization sample* *Mildly elevated =T scores of 55–59 were obtained by <25% of the standardization sample* *Moderately elevated = T scores of 60–69 and were obtained by <15% of the standardization sample* *Extremely elevated = T scores >70 were obtained by <5% of the standardization sample*	Ages 7–16 years, completed by subjects
Beery-Buktenica Development Test-6 (23)	Visual-Motor Integration (VMI) *The VMI assesses the visual-perceptual skills (a child’s ability to make sense of and interpret what they see) and visual-motor skills (ability to translate a visual image or plan into a motor action)*	All ages, completed by subjects
BASC-3 (24)	Emotional/behavioral adjustment *The following composite scores were analyzed: (a) behavioral symptoms that assess levels of anxiety, depression, attention problems, hyperactivity, and aggression* *(b) internalizing problems that consist of a child’s emotional of psychological state and typically include depression, anxiety, and somatic complaints* *(c) externalizing problems that are manifested in a child’s outward behavior and reflect negatively on the external environment (e.g., disruptive, hyperactive, and aggressive behavior)*	All ages, completed by parents only
BRIEF-2 (25)	Executive function (self- and parent report) *Assesses the child’s everyday behaviors associated with executive function at home and at school. There are three components of the test: the behavior regulation index, the emotion regulation index, and the cognitive regulation index. There is an overall summary score, the global executive composite score*	Ages 11–16 years, completed by both subjects and parents Ages 6–10 years, this was completed by parents only
CVLT-C (26)	Verbal learning and memory *Assesses long-term memory based on the ability to recall words from a list*	All ages, completed by subjects
D-KEFS Tower Test and Trail Making Test) (27)	Executive function *A collection of interrelated functions responsible for guiding, directing, and managing cognitive emotional and behavioral responses*	Ages 8–16 years, completed by subjects
WISC-V (28)	Overall intellectual function *The FSIQ consists of subtests measuring verbal comprehension, visual spatial, fluid reasoning, working memory, and processing speed*	All ages, completed by subjects

### Statistical analysis

Continuous variables were summarized by calculating the mean, SD, median, and range. Categorical variables were described using frequencies and percentages. ND testing results were also dichotomized based on clinically relevant cut points and the frequencies in each category summarized and compared with expectation in a normative population using a one-sample binomial test.

Univariate analyses using two independent sample *t* tests or ANOVA were performed to examine the associations between ND outcomes and the key variables of trigger for diagnosis, mutation group, and conditioning intensity. We considered a P value of <0.01 to be statistically significant. Multiple linear regression using stepwise variable selection was used to develop prognostic factor models for primary endpoints.

Multivariate analysis included: race and ethnicity, gender, mutation group, donor type, conditioning intensity, GVHD, history of chronic GVHD, transplant type, age of child at time of testing, self-reported annual household, self-reported highest level of education of parents, specific infections at baseline, use of mechanical ventilation, trigger for diagnosis, languages other than English or French spoken in the home, height Z score, and weight Z score.

All statistical analyses were conducted using SAS 9.4 (SAS Institute).

### Online supplemental material

Online supplemental tables are available to describe our study population and the potentially eligible population (Table S1) and variables for the five subjects who had significantly low-test scores (Table S2).

## Supplementary Material

Table S1 shows generalizability of sample compared to original source of potential participants.

Table S2 shows characteristics of five patients who had significantly low overall IQ scores.
