# Supplementary material for: Neurodevelopmental outcomes following hematopoietic cell transplantation for patients with severe combined immunodeficiency (SCID): A PIDTC study
Source: J Hum Immun. 2025 Dec 17;2(1):e20250163. doi: 10.70962/jhi.20250163 (PMC12829746; doi:10.70962/jhi.20250163)
Supplement: Table S1 — shows generalizability of sample compared to original source of potential participants. [file jhi_20250163_tables1.docx]

**Supplemental Table 1: Generalizability of Sample Compared to Original Source of Potential Participants**

| **Variables** | **Potential Subjects at Participating Centers  n=162 (col %)** | **Subjects Participating * n=79 (col %)** | **DID NOT PARTICIPATE**  **Not Included in Study n=83 (col %)** | **Comparison of Subjects Participating to Potential Subjects P Value** |
| --- | --- | --- | --- | --- |
| **Transplant group** |  |  |  | *0.076 ^C+^* |
| ERT-HCT | 6 (3.7) | 5 (6.3) | 1 (1.2) |  |
| GT | 10 (6.2) | 7 (8.9) | 3 (3.6) |  |
| HCT | 146 (90.1) | 67 (84.8) | 79 (95.2) |  |
| **Sex** |  |  |  | *0.371 ^C^* |
| Female | 46 (28.4) | 25 (31.6) | 21 (25.3) |  |
| Male | 116 (71.6) | 54 (68.4) | 62 (74.7) |  |
| **Race** |  |  |  | *0.414 ^C+^* |
| American Indian/Alaska native | 6 (3.7) | 4 (5.1) | 2 (2.4) |  |
| Asian/Pac Islander | 9 (5.6) | 4 (5.1) | 5 (6.0) |  |
| Black or African American | 22 (13.6) | 7 (8.9) | 15 (18.1) |  |
| Unknown or Not Reported | 23 (14.2) | 13 (16.5) | 10 (12.0) |  |
| White | 102 (63.0) | 51 (64.6) | 51 (61.4) |  |
| **Ethnicity** |  |  |  | *0.665 ^C^* |
| Hispanic, Latino, or Spanish origin | 46 (28.4) | 21 (26.6) | 25 (30.1) |  |
| Not Hispanic, Latino, or Spanish origin | 97 (59.9) | 47 (59.5) | 50 (60.2) |  |
| Unknown or not reported | 19 (11.7) | 11 (13.9) | 8 (9.6) |  |
| **Mutation** |  |  |  | *0.094 ^C+^* |
| ADA | 15 (9.3) | 10 (12.7) | 5 (6.0) |  |
| DCLRE1C | 4 (2.5) | 4 (5.1) | 0 (0.0) |  |
| IL2RG/JAK3 | 75 (46.3) | 34 (43.0) | 41 (49.4) |  |
| IL7R/CD3D | 16 (9.9) | 10 (12.7) | 6 (7.2) |  |
| Other/Unknown | 23 (14.2) | 10 (12.7) | 13 (15.7) |  |
| RAG 1/2 | 29 (17.9) | 11 (13.9) | 18 (21.7) |  |
| **Final Trigger** |  |  |  | *0.562 ^C^* |
| FH | 30 (18.8) | 12 (15.4) | 18 (22.0) |  |
| Infection/clin | 52 (32.5) | 26 (33.3) | 26 (31.7) |  |
| NBS | 78 (48.8) | 40 (51.3) | 38 (46.3) |  |
| Missing | 2 | 1 | 1 |  |
| **Type of Transplant** |  |  |  | *0.142 ^C+^* |
| Autologous | 10 (6.2) | 7 (8.9) | 3 (3.6) |  |
| HLA-identical sibling | 16 (9.9) | 6 (7.6) | 10 (12.0) |  |
| HLA-matched URD /other relative | 54 (33.3) | 28 (35.4) | 26 (31.3) |  |
| HLA-mismatched relative | 40 (24.7) | 23 (29.1) | 17 (20.5) |  |
| HLA-mismatched unrelated | 42 (25.9) | 15 (19.0) | 27 (32.5) |  |
| **Conditioning** |  |  |  | ***<0.001*** *^C^* |
| None/IS | 56 (34.6) | 40 (50.6) | 16 (19.3) |  |
| RIC/MAC | 106 (65.4) | 39 (49.4) | 67 (80.7) |  |
| **Product type (first CT)** |  |  |  | *0.065 ^C+^* |
| BM | 76 (46.9) | 36 (45.6) | 40 (48.2) |  |
| Bone marrow CD34+ cells | 10 (6.2) | 7 (8.9) | 3 (3.6) |  |
| PBSC | 38 (23.5) | 23 (29.1) | 15 (18.1) |  |
| UCB | 38 (23.5) | 13 (16.5) | 25 (30.1) |  |
| **GVHD (first HCT)** |  |  |  | *0.828 ^C^* |
| 1 CD34 selection +/- TCD | 52 (32.1) | 28 (35.4) | 24 (28.9) |  |
| 2 IS + ATG/Campath | 59 (36.4) | 27 (34.2) | 32 (38.6) |  |
| 3 IS only | 35 (21.6) | 16 (20.3) | 19 (22.9) |  |
| 4 None | 16 (9.9) | 8 (10.1) | 8 (9.6) |  |
| **Year of First CT** |  |  |  | *0.375 ^W^* |
| Median (min - max) | 2013.0 (2005.0 - 2018.0) | 2014.0 (2005.0 - 2018.0) | 2013.0 (2006.0 - 2017.0) |  |
| ^+^Exact test; ^T^T-test; ^W^Wilcoxon rank-sum test; ^C^Chi-square test; ^*^ 79 subjects includes those with ADA deficiency | | | | |
|  | | | | |
