# Supplementary material for: Neurodevelopmental outcomes following hematopoietic cell transplantation for patients with severe combined immunodeficiency (SCID): A PIDTC study
Source: J Hum Immun. 2025 Dec 17;2(1):e20250163. doi: 10.70962/jhi.20250163 (PMC12829746; doi:10.70962/jhi.20250163)
Supplement: Table S2 — shows characteristics of five patients who had significantly low overall IQ scores. [file jhi_20250163_tables2.docx]

**Supplemental Table 2:**

**Characteristics of Five Patients Who Had Significantly Low Overall IQ Scores**

| **Subject** | **Gender/ Race/ Ethnicity** | **Genotype** | **Trigger for Diagnosis** | **Conditioning^*^** | **Infection** | **Family Income** | **Additional Comments** |
| --- | --- | --- | --- | --- | --- | --- | --- |
| 1 | M/ White | Unknown | Infection | MAC | Pre-Transplant: CMV viremia  Varicella, Herpes | >150K | Mechanical ventilation, developmental delay at 6 months of age |
| 2 | M/ White | IL2RG | Infection | Transplant 1:  Gene tx, no conditioning  Transplant 2:  Gene tx, IS alone  Transplant 3:  MUD: RIC | Pre-Transplant:  E coli meningitis, CMV viremia  Seizure disorder | Prefer not to answer | na |
| 3 | F/ White/Hispanic | IL2RG | NBS | RIC | Staphylococcus cellulitis  HSV stomatitis  (resolved after transplant) | Did not answer | Indigenous family, parents did not speak either English or Spanish |
| 4 | M/ White | Unknown | Infection | None/ IS | Pre-Transplant:  PJP /CMV  Post-Transplant: Adenovirus and varicella | 50-75K | Developmental Delay reported at 1 year |
| 5 | M/ White | Unknown | Infection | None/IS | PJP | Prefer not to answer | na |

* Definitions MAC: myeloablative conditioning; RIC reduced intensity conditioning; IS immunosuppression; HSV: herpes simpex virus; PJP: pneumocystis jirovecii; CMV: cytomegalovirus; NA: not available
